# Small Bowel Pleomorphic Liposarcoma: A Rare Cause of Gastrointestinal Bleeding

**DOI:** 10.1155/2014/391871

**Published:** 2014-08-04

**Authors:** Simon Nennstiel, Martin Mollenhauer, Christoph Schlag, Valentin Becker, Bruno Neu, Norbert Hüser, Ralf Gertler, Roland M. Schmid, Stefan von Delius

**Affiliations:** ^1^II. Medizinische Klinik und Poliklinik, Klinikum rechts der Isar der Technischen Universität München, Ismaninger Straße 22, 81675 München, Germany; ^2^Institut für Allgemeine Pathologie und Pathologische Anatomie, Klinikum rechts der Isar der Technischen Universität München, Ismaninger Straße 22, 81675 München, Germany; ^3^Chirurgische Klinik und Poliklinik, Klinikum rechts der Isar der Technischen Universität München, Ismaninger Straße 22, 81675 München, Germany

## Abstract

In this case report we present a 60-year-old male patient with overt midgastrointestinal bleeding of a primary ileal pleomorphic liposarcoma diagnosed by video capsule endoscopy (VCE). Clinical work-up for final diagnosis and the pathological background of this uncommon tumorous entity of the small bowel will be discussed in this paper.

## 1. Introduction

Severe gastrointestinal bleeding is a medical emergency situation, asking for rapid diagnosis and treatment. In case of suspected mid-gastrointestinal bleeding, following negative gastroscopy and colonoscopy, video capsule endoscopy (VCE) is known to be an effective examination for the diagnosis of the hemorrhagic site [1].

In this case report we present the clinical and the pathological findings of a rare tumor of the small bowel that lead to severe gastrointestinal bleeding—a primary ileal pleomorphic liposarcoma.

## 2. Case Presentation

We report a 60-year-old male patient who was hospitalized due to gastrointestinal bleeding. The patient initially presented to the referring hospital with melaena and symptomatic anemia (hemoglobin 6.3 g/dL). Similar episodes of melaena, however non-Hb-relevant, were reported by the patient since 2009. The patient's medical history showed chronic renal failure, hypothyreosis, and hypertension. The patient was on regular therapy with acetylsalicylic acid (100 mg/day), thyroxin, blood pressure medications, and a statin.

Gastroscopy and colonoscopy showed no source of hemorrhage; however, fresh blood was detected in the terminal ileum.

At time of initial admission to our hospital, the patient's hemoglobin value was still low (6.7 g/dL), although he had already received two packed red blood cells (RBCs). The patient received four more RBCs during the first three days of hospital stay; afterwards Hb value proved to be stable and no more RBCs were needed.

Following admission to our hospital, we conducted emergency single-balloon enteroscopy (Olympus SIF Q180) of the proximal small bowels, reaching into the distal jejunum, without identification of the source of hemorrhage. In addition, colonoscopy with deep penetration into the ileum (approximately 30 cm) was repeated without detection of the hemorrhagic site. There was no evidence of fresh or old blood in the ileum or colon.

**Figure 1:**
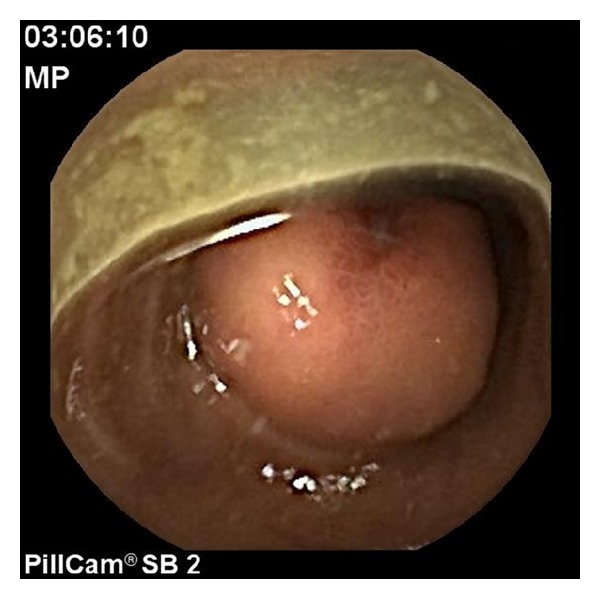
Video capsule endoscopy showing a centrally exulcerated tumor in the ileum.

Subsequent video capsule endoscopy (Pillcam SB2; Given Imaging, Israel) was not able to detect the bleeding site due to insufficient bowel cleansing, because of feces and hematin in the middle/distal small bowels, even though laxative preparation was adequate for prior colonoscopy. After additional laxative bowel cleansing (PEG, Oralav, B Braun, Germany) of the intestines and after ceasing of hemorrhagic signs, second capsule endoscopy (day 7 after referral to our hospital) revealed a centrally exulcerated, subepithelial tumorous lesion with luminal growth in the ileum (Figure 1). Again, capsule endoscopy showed intestinal defilement before and past the tumorous lesion.

Supplementary contrast-enhanced computed tomography (CT) for staging purpose was not able to detect the tumorous lesion of the small bowels—there were no signs of local or distal metastases.

The patient was referred for surgery. Following median laparotomy, the tumor could be palpated approximately 40 cm orally to the ileocecal valve. Second a Meckel's diverticulum was found aborally to the tumor without signs of inflammation. Ileum-segment resection (30 cm) and side-to-side ileoileostomy were performed. Seven days postoperatively the patient could be discharged without any residual impairment or further blood loss.

**Figure 2:**
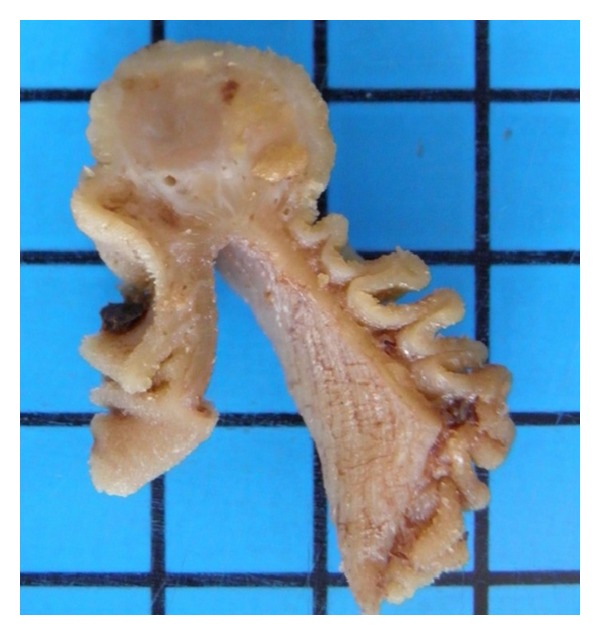
Fixated gross section of the polypoid submucosal tumor protruding the mucosa.

**Figure 3:**
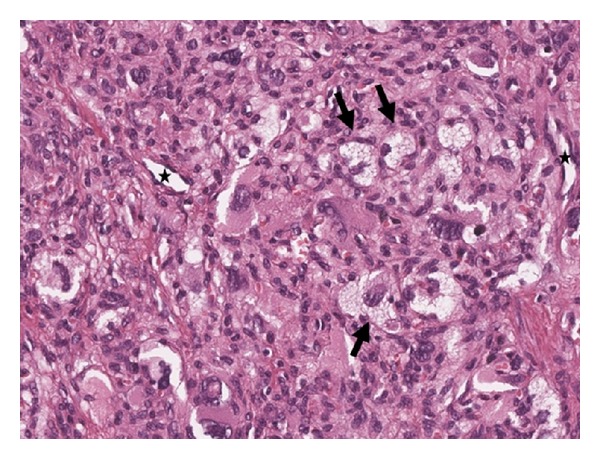
Histology (300x, H & E stain) showed a solid tumor with interspersed lipoblasts (arrow) and prominent small vessels (∗).

Histologically the small tumor (max. diameter 1.8 cm) showed well-defined borders and consisted of a mostly solid and haphazard proliferation of undifferentiated cells in a mostly solid, partly pericytoma-like growth pattern. The tumor emerged from the submucosa with polypoid protrusion of the mucosa (Figure 2), without penetration of intestinal lumen. Bleeding was caused by mucosal damage, caused by the pressure due to the tumor growth. The tumor cells were highly pleomorphic with 8 mitoses/10 HPF. Lipoblasts with hyperchromatic nuclei and wide and vacuolated cytoplasm were interspersed in the tumor (Figure 3). Immunohistochemically the tumor cells focally expressed S-100; none of the other examined markers including cytokeratin, synaptophysin, chromogranin A, and DOG-1 showed positive immunoreaction. In conclusion the tumor was diagnosed as a pleomorphic liposarcoma with hemangiopericytoma-like growth pattern.

## 3. Discussion

In general, liposarcomas are one of the most common soft-tissue sarcomas in adults. However, manifestation in the small bowel is rare [2, 3]. The pleomorphic subtype of liposarcomas is the least common, comprising less than 15% of all liposarcomas. Predilection sites of pleomorphic liposarcomas are the retroperitoneum, the deep somatic soft tissues of the extremities, and the skin/subcutis [4]. Experience with small intestine liposarcoma is limited to case reports. Clinical findings at time of initial diagnosis are often nonspecific, including intussusceptions [5], symptoms similar to appendicitis [6], intestinal obstruction [7], or peritonitis due to perforation of the small bowels [8]. Severe small bowel hemorrhage is an uncommon complication of small intestinal liposarcoma. In fact, to our knowledge this case report presents the first case of severe small intestinal hemorrhage due to pleomorphic liposarcoma.

In the detection of small bowel hemorrhage, video capsule endoscopy (VCE) and balloon-assisted enteroscopy (BAE) are techniques with a similar diagnostic yield [9]. However, VCE offers higher rates of complete small bowel visualization compared to BAE [10]. In addition, BAE is known to be technically challenging and time consuming, especially when performed by the retrograde approach [11]. In contrast, VCE is easy to apply. The disadvantage of VCE is the inability for therapeutic intervention and the possible complication of capsule retention [12]. Additionally, in case of overt bleeding it can be difficult to identify the exact source of bleeding because of limited visualization caused by the blood [1, 10]. VCE shows the highest diagnostic yield when performed as close as possible to the bleeding episode [13]. Therefore, early “emergency” application has been proposed in severe obscure-overt gastrointestinal bleeding [1].

In clinical practice, VCE is usually used as the first diagnostic approach to the small bowels, enabling localization and assessment of the bleeding site, and can then be followed by BAE for endoscopical haemostasis [14]. Hereby, VCE can deliver important information whether to prefer an ante- or retrograde BAE. Surgery might also become a therapeutic option if a tumorous lesion is suspected by VCE. However VCE's main limitation—the lack of therapeutical intervention—has to be considered particularly in severe bleeding.

Therefore, in this case of severe hemorrhage, we decided to perform antegrade BAE as the primary approach to the small bowel in order to achieve rapid endoscopical haemostasis. In the retrospective, and this underlines the importance of VCE in severe bleeding, VCE performed prior to balloon enteroscopy could have predicted the bleeding site and antegrade balloon-assisted enteroscopy would not have been necessary. Moreover, retrograde balloon-assisted enteroscopy or surgery could have been performed at an early stage.

Retrograde balloon-assisted enteroscopy was not performed in this case, because the tumor was already diagnosed by VCE and the indication for primary surgery was decided, based on estimated intraoperative identifiability of the tumor. However, some (particularly small) tumors might not be easily palpated during surgery. Thus, retrograde BAE after VCE might have been useful in order to mark the tumor for scheduled surgery. Since the liposarcoma was located only approximately 40 cm orally to the ileocecal valve, the effort for BAE could be regarded justifiable to eventually ensure a safer subsequent surgical resection.

Complete surgical resection is the best treatment of liposarcomas of the small intestine, given that it is diagnosed at an early stage with no evidence of distant metastasis [7]. In our case the complete tumor was removed by segmental resection of about 30 cm of the ileum prior to a definite pathological diagnosis. Since tumor staging showed no evidence for distant metastasis in the presented patient, prognosis was suspected to be good. Nevertheless, further follow-up of the patient is mandatory, since sufficient data concerning the prognosis of this rare tumor is sparse. Subsequent distal metastases of pleomorphic liposarcomas in different locations have been described especially in the lungs [15].

In conclusion, the proper application of video capsule endoscopy is an important method for the diagnosis of small bowel hemorrhage. In the current case VCE revealed the uncommon finding of a primary liposarcoma of the small bowel. Surgical resection was conducted and the patient is expected to have a good prognosis.
